# Supplementary figures and images for: Identification of potential molecular mimicry in pathogen-host interactions
Source: PeerJ. 2023 Nov 7;11:e16339. doi: 10.7717/peerj.16339 (PMC10637249; doi:10.7717/peerj.16339)

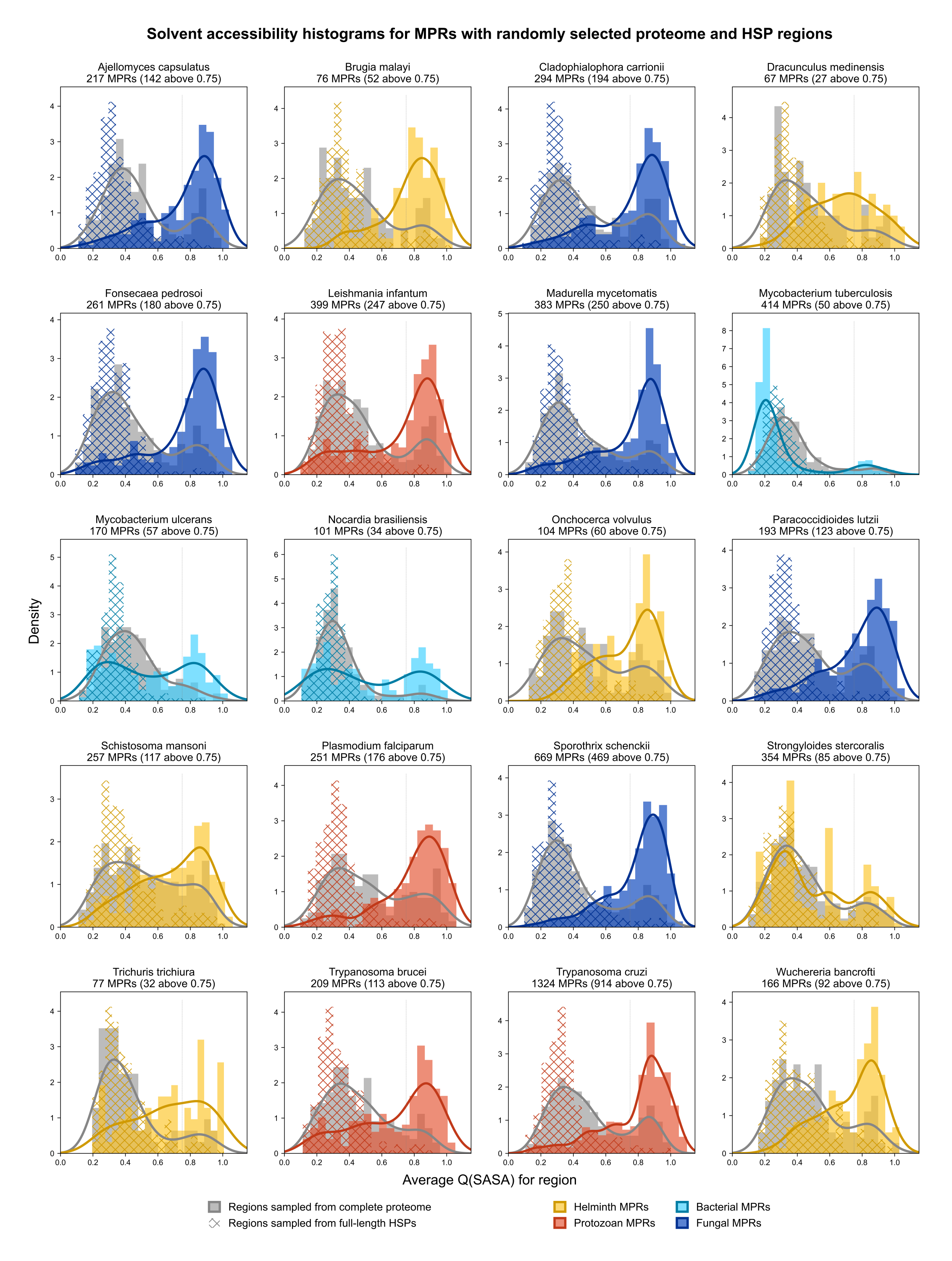

Supplement: Supplemental Information 2 — Histograms/KDE plots for average solvent accessibility of MHRs with equivalent-length regions randomly selected from the complete pathogen proteome (grey) and from full-length protein HSPs (hatched). [file peerj-11-16339-s002.png]

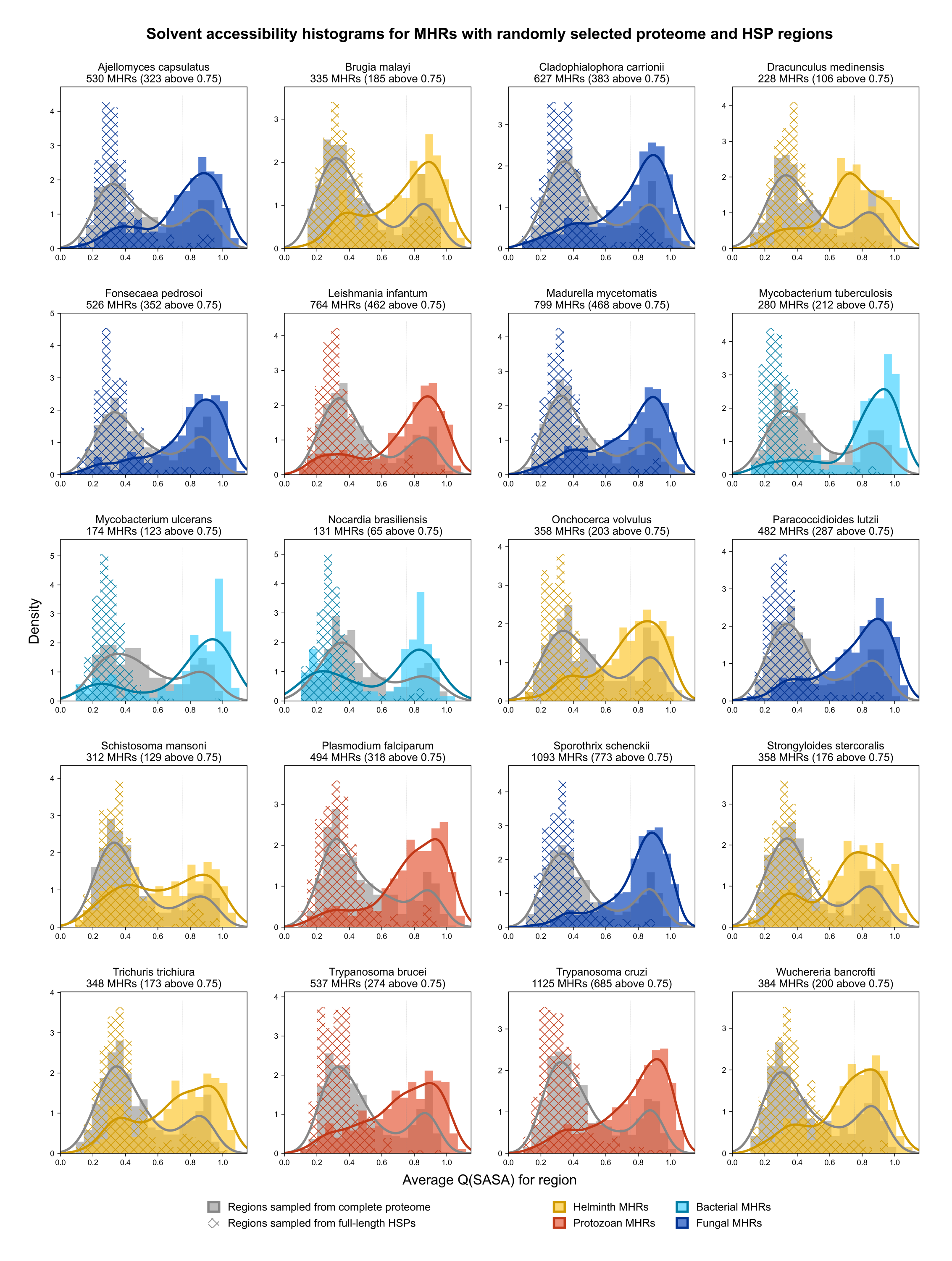

Supplement: Supplemental Information 3 — Histograms/KDE plots for average solvent accessibility of MPRs with equivalent-length regions randomly selected from the complete human proteome (grey) and from full-length protein HSPs (hatched). [file peerj-11-16339-s003.png]
